# Supplementary material for: Fluorocarbosilane-Based Protective Coatings for Concrete
Source: Materials (Basel). 2022 Aug 30;15(17):5994. doi: 10.3390/ma15175994 (PMC9457494; doi:10.3390/ma15175994)
Supplement: Supplementary file 1 [file materials-15-05994-s001.zip › materials-1842538-supplementary.pdf]

Fluorocarbosilane-based protective coatings for concrete

Supporting Information

Karol Szubert, Agnieszka Dutkiewicz, Marek Nowicki, Hieronim Maciejewski

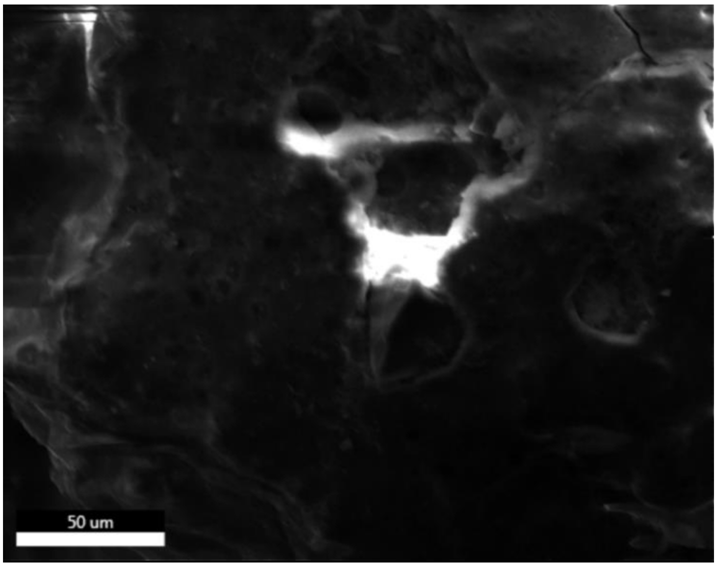

Back-scattered electron image of unmodified concrete

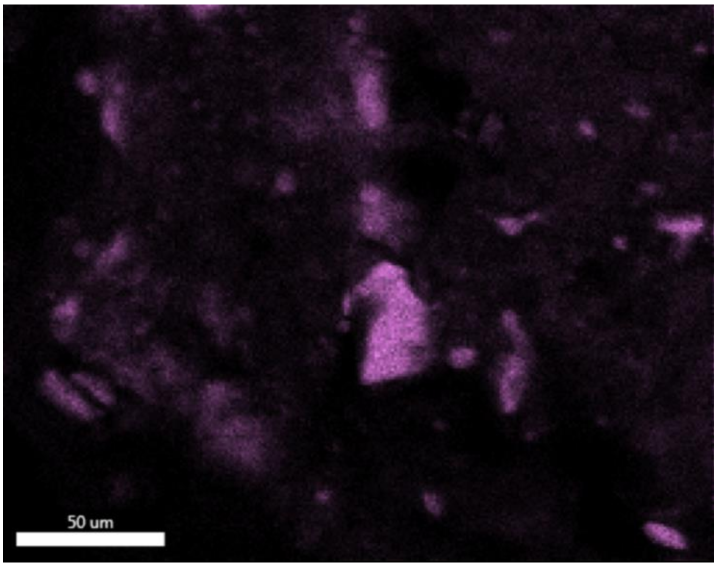

Element map of Si in unmodified concrete

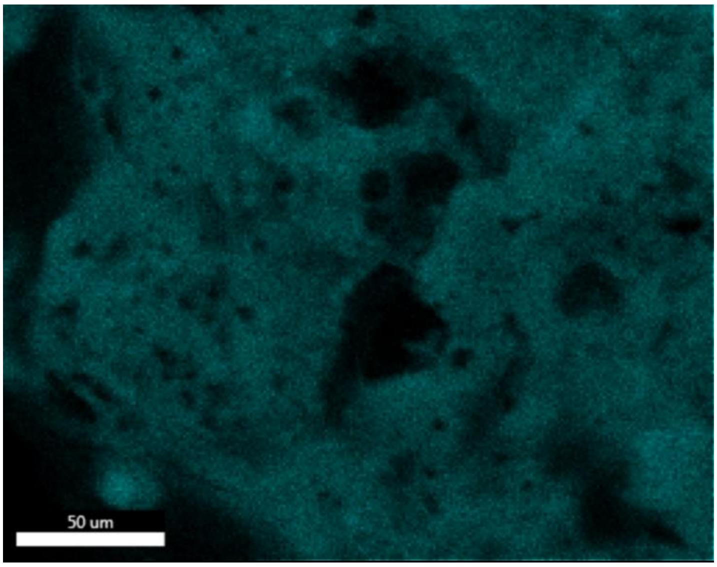

Element map of Ca in unmodified concrete

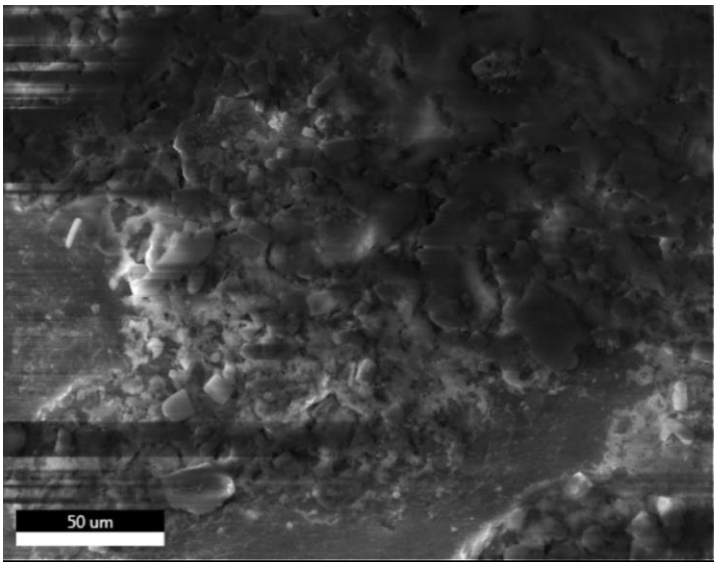

Back-scattered electron image of OFTES-coated samples **F1**

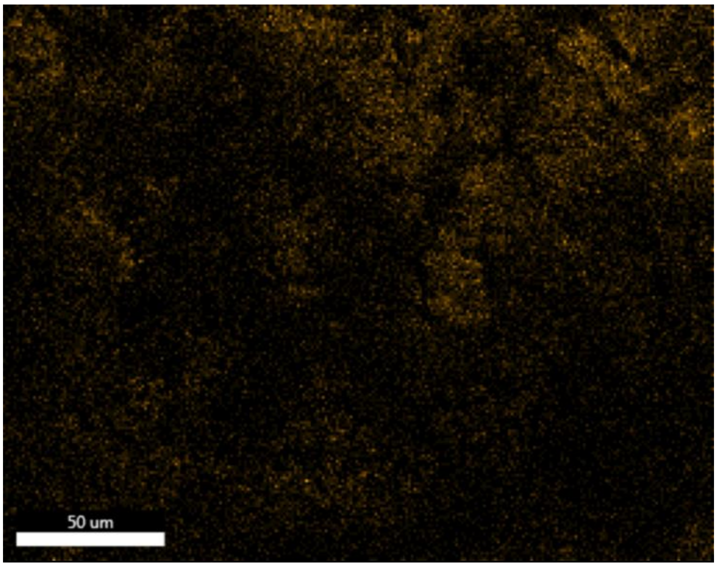

Element map of F in OFTES-coated samples **F1**

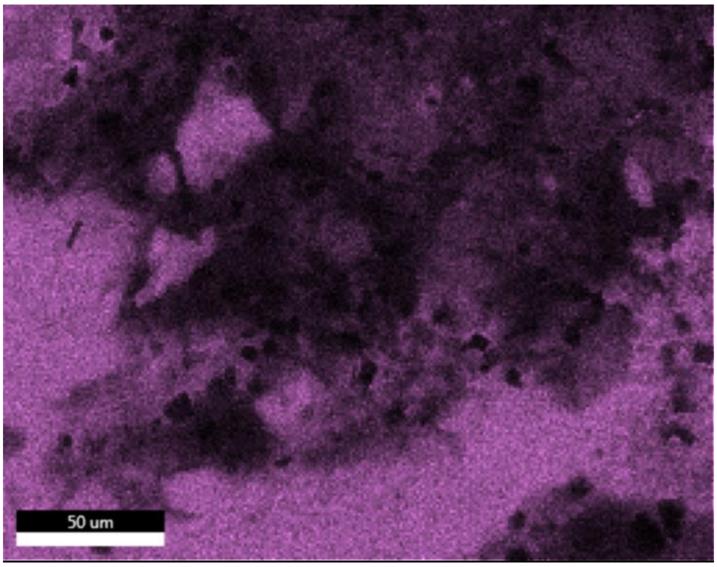

Element map of Si in OFTES-coated samples **F1**

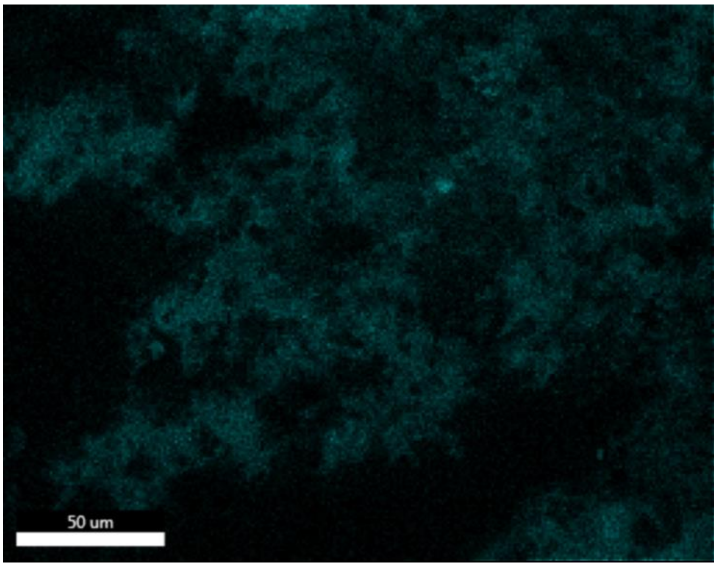

Element map of Ca in OFTES-coated samples **F1**

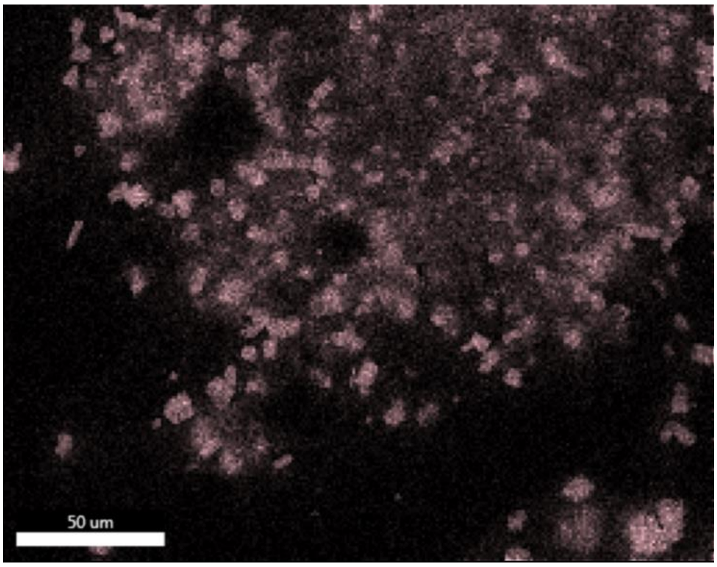

Element map of Cl in OFTES-coated samples **F1**

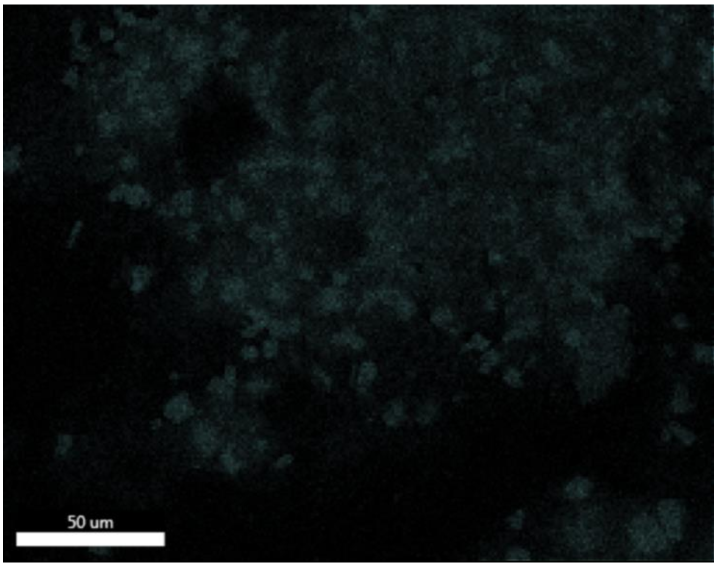

Element map of K in OFTES-coated samples **F1**

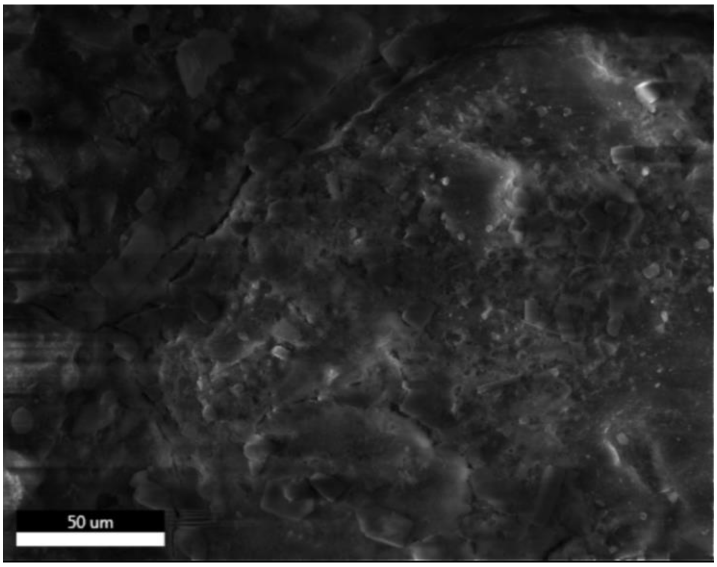

Back-scattered electron image of OFTES-coated samples **F2.5**

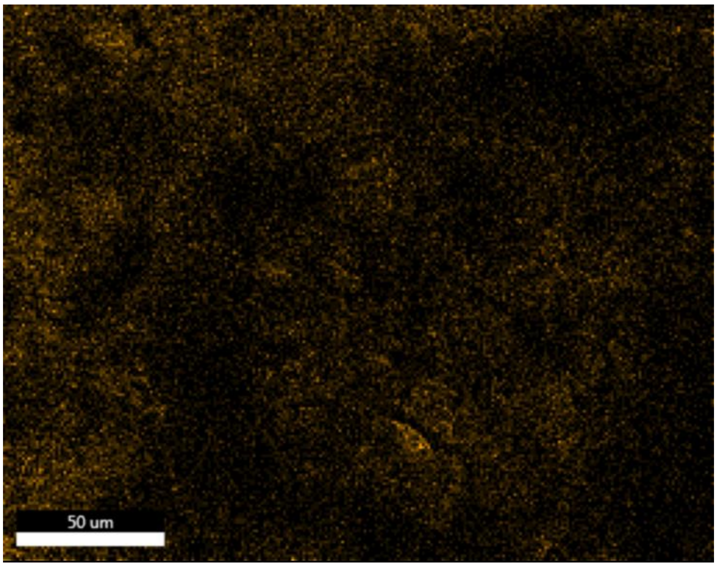

Element map of F in OFTES-coated samples **F2.5**

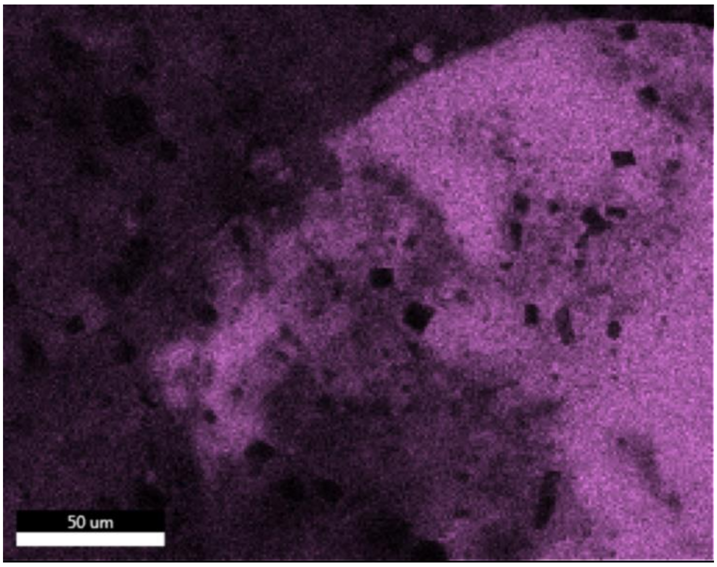

Element map of Si in OFTES-coated samples **F2.5**

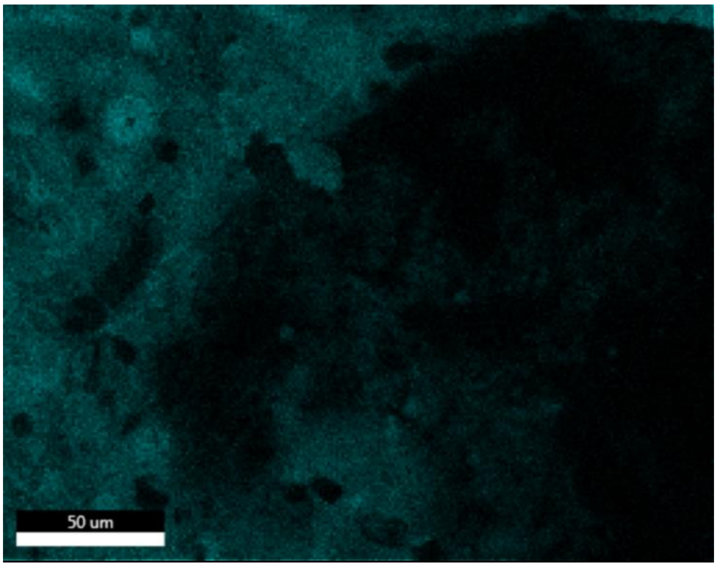

Element map of Ca in OFTES-coated samples **F2.5**

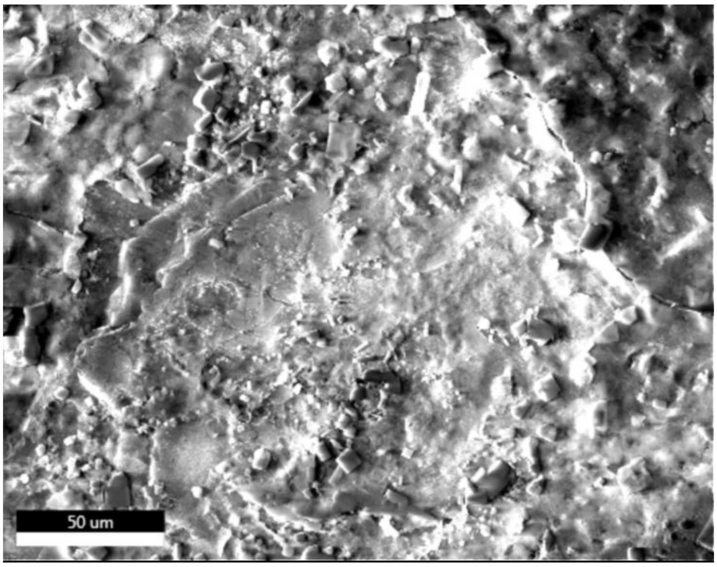

Back-scattered electron image of OFTES-coated samples **F5**

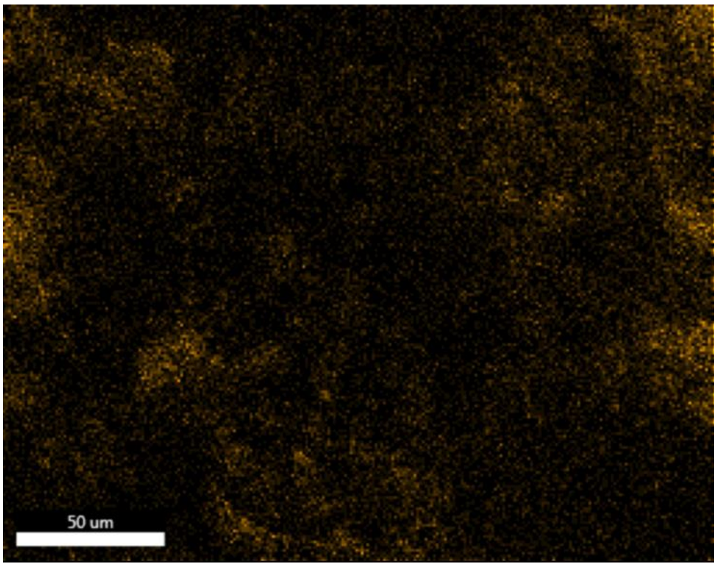

Element map of F in OFTES-coated samples **F5**

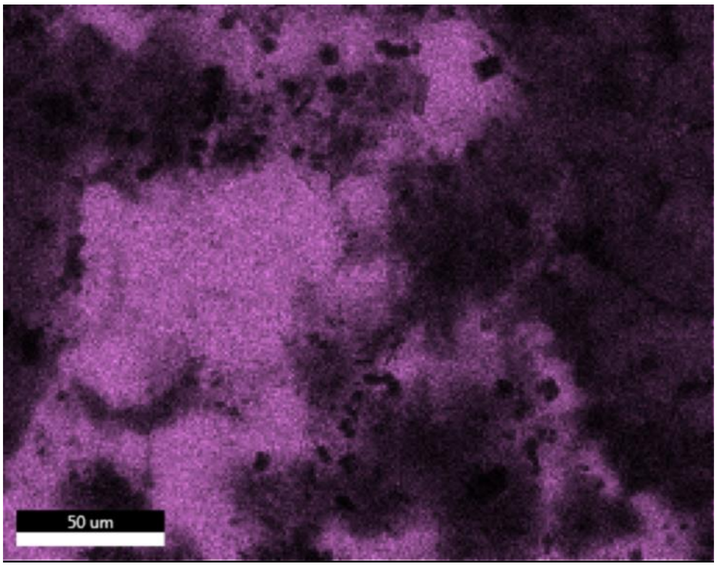

Element map of Si in OFTES-coated samples **F5**

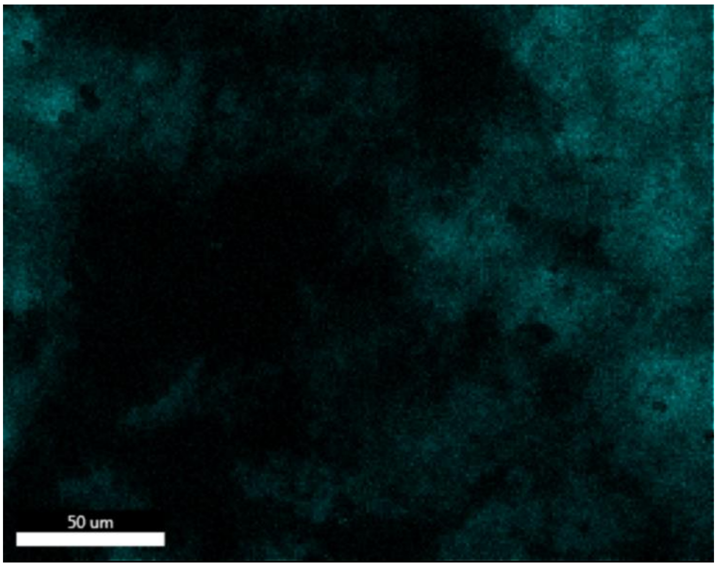

Element map of Ca in OFTES-coated samples **F5**

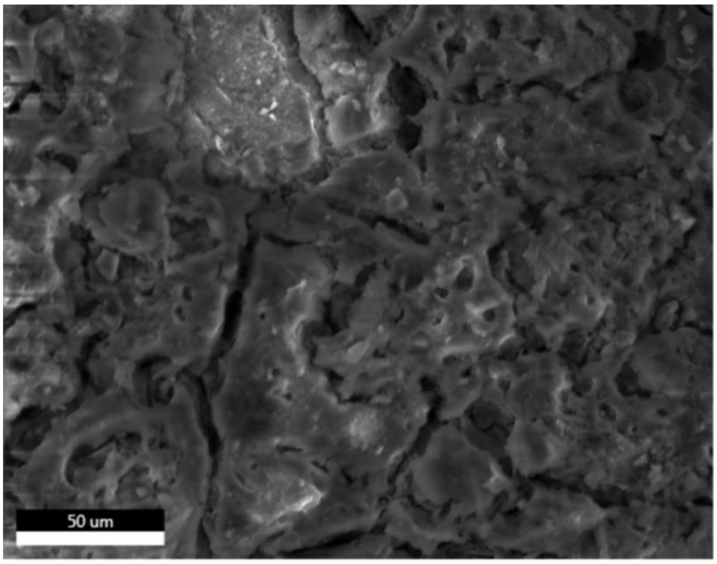

Back-scattered electron image of OFTES-coated samples **F1_4**

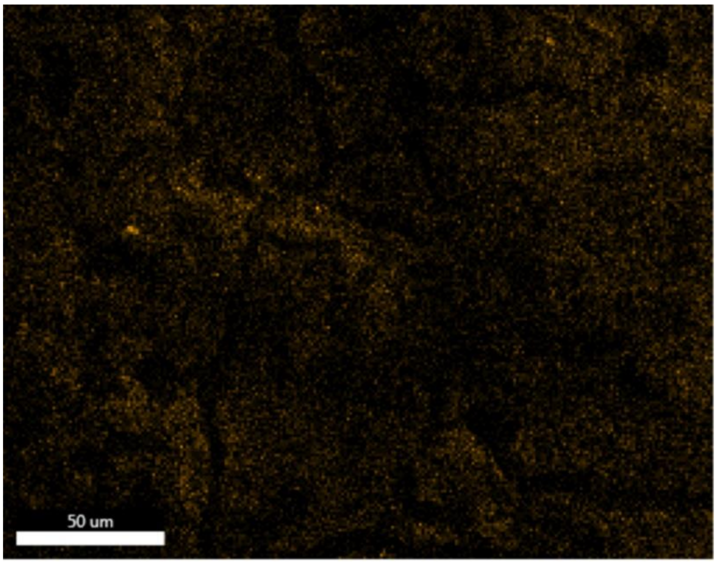

Element map of F in OFTES-coated samples **F1_4**

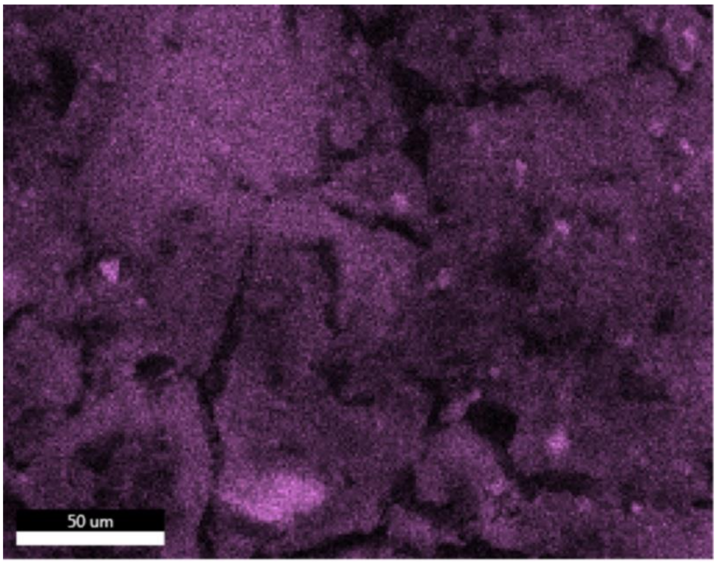

Element map of Si in OFTES-coated samples **F1_4**

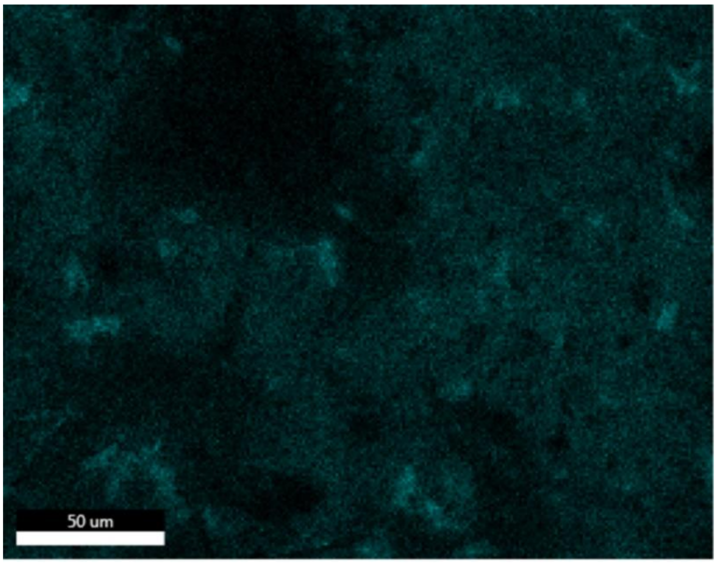

Element map of Ca in OFTES-coated samples **F1_4**

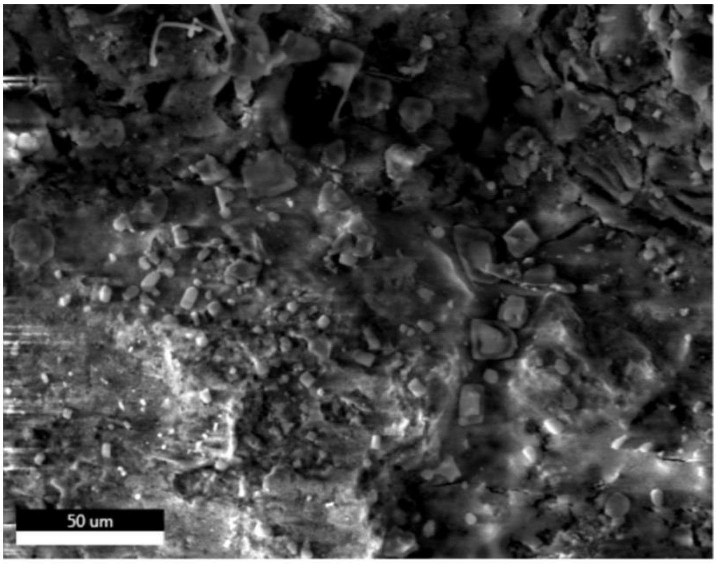

Back-scattered electron image of OFTES-coated samples **F2.5_2.5**

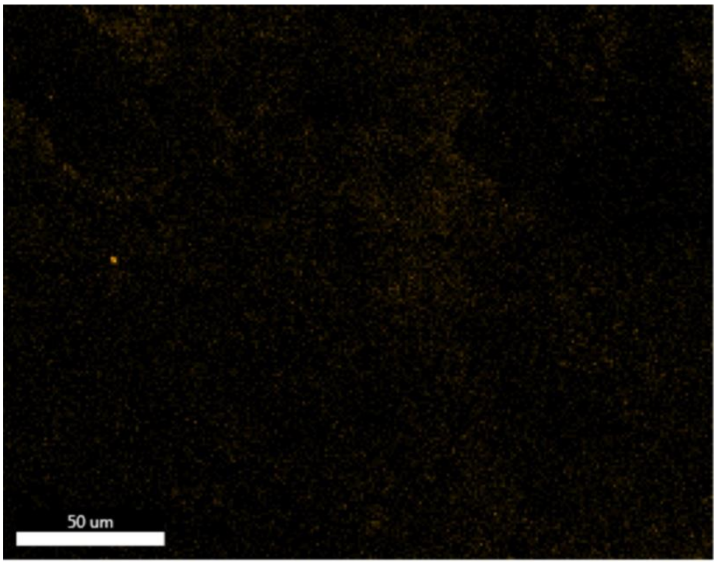

Element map of F in OFTES-coated samples **F2.5_2.5**

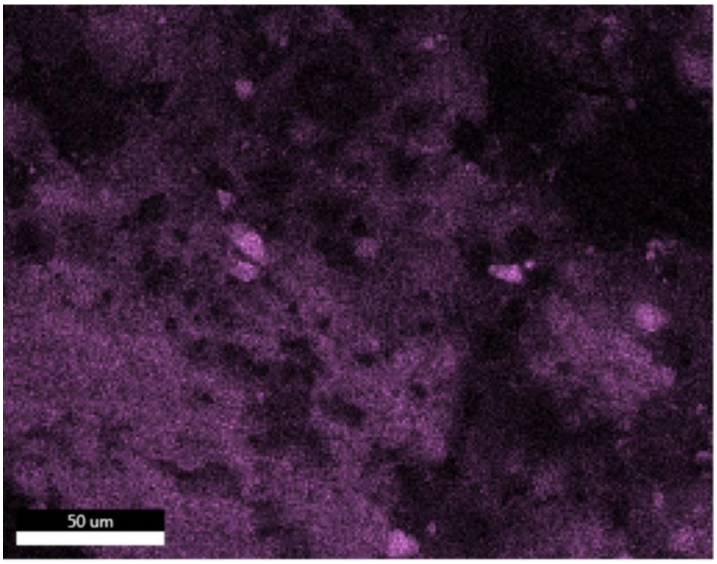

Element map of Si in OFTES-coated samples **F2.5_2.5**

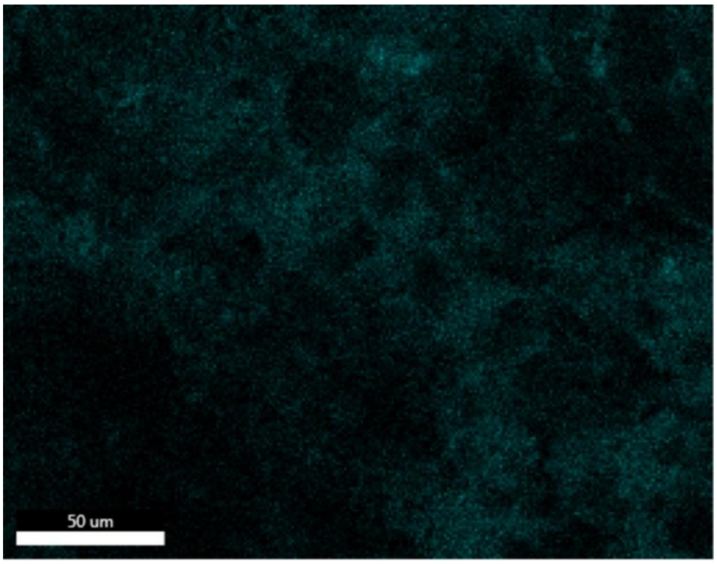

Element map of Ca in OFTES-coated samples **F2.5_2.5**

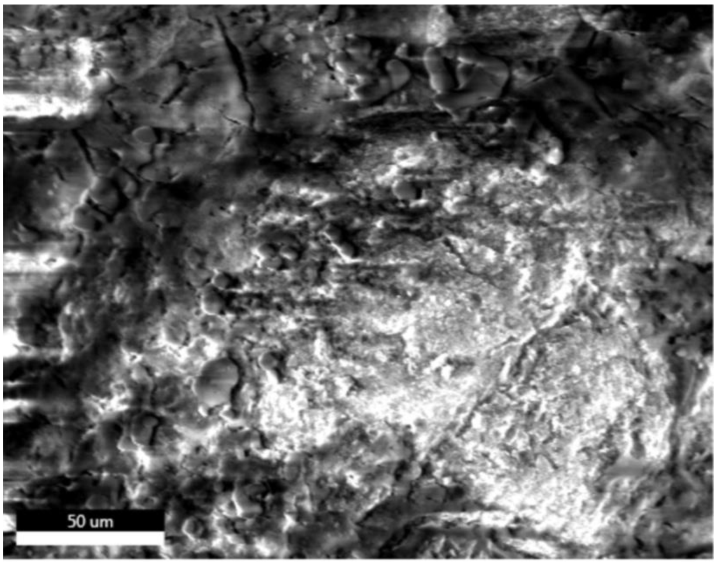

Back-scattered electron image of OFTES-coated samples **F4_1**

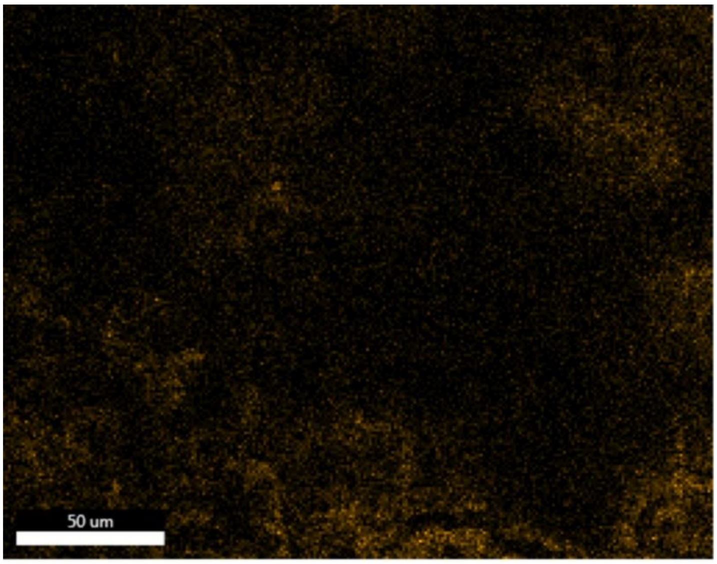

Element map of F in OFTES-coated samples **F4_1**

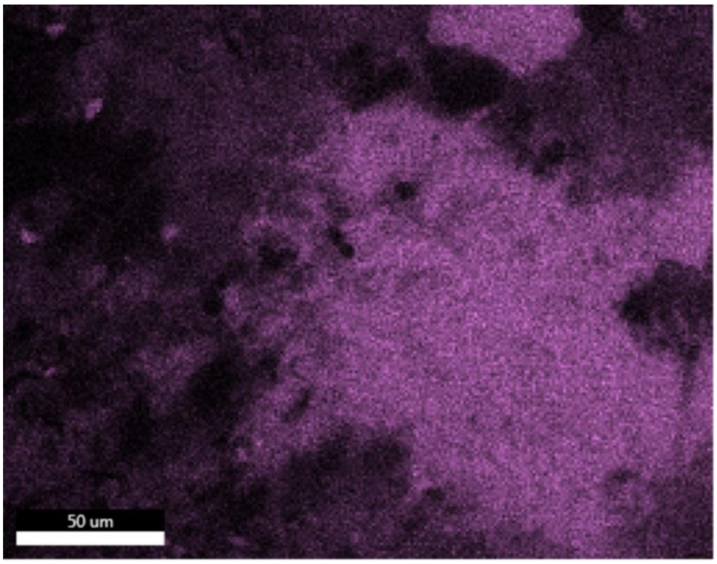

Element map of Si in OFTES-coated samples **F4_1**

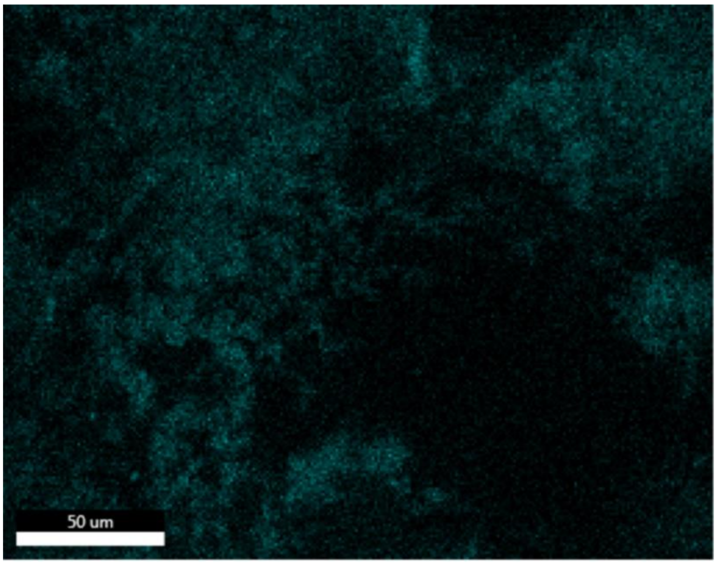

Element map of Ca in OFTES-coated samples **F4_1**

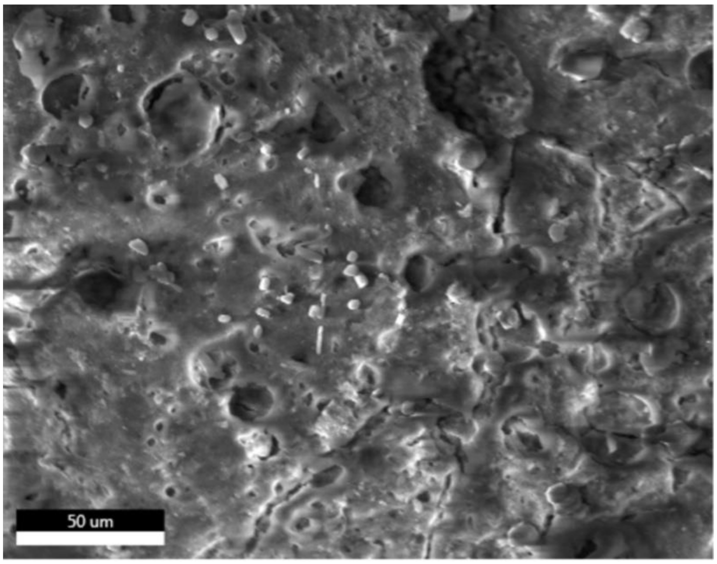

Back-scattered electron image of OFTES-coated samples **F5_5**

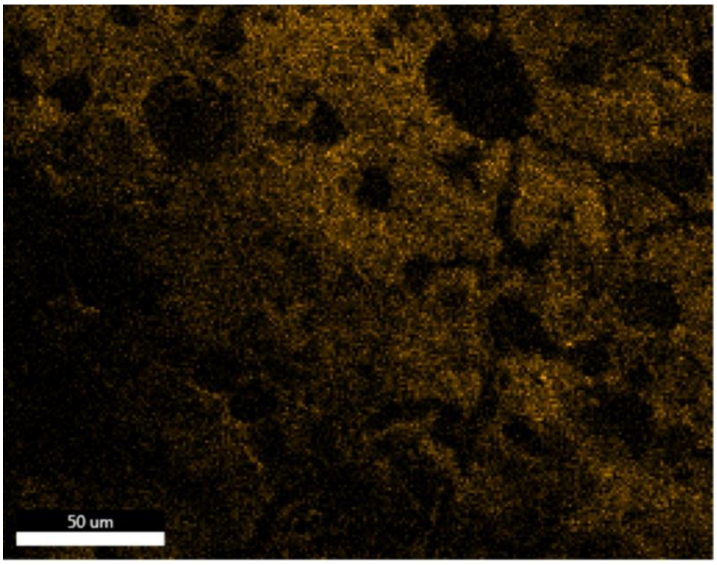

Element map of F in OFTES-coated samples **F5_5**

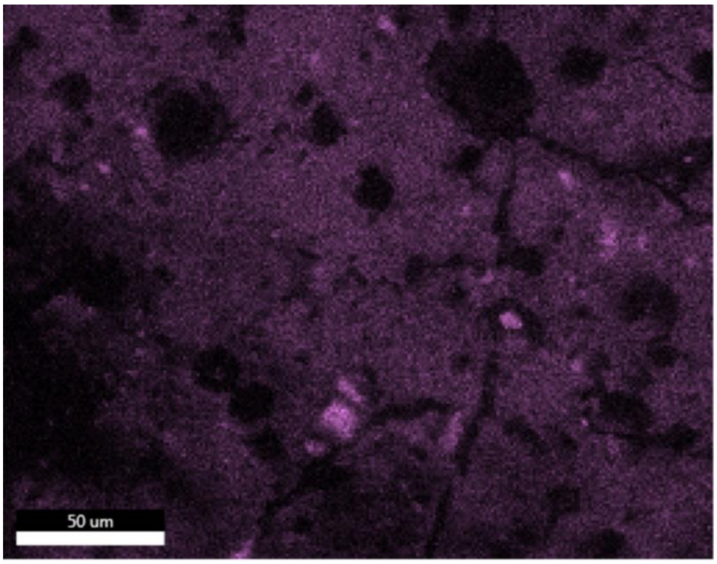

Element map of Si in OFTES-coated samples **F5_5**

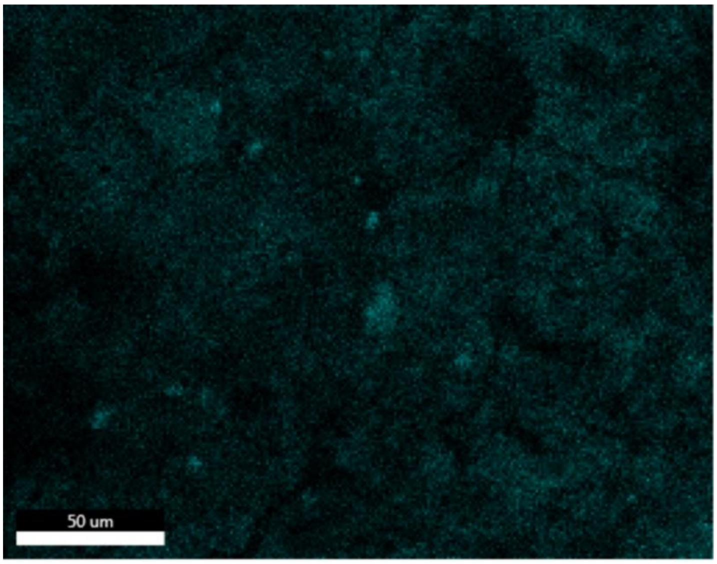

Element map of Ca in OFTES-coated samples **F5_5**
